# Imaging Radiation-Induced Gastrointestinal, Bone Marrow Injury and Recovery Kinetics Using ^18^F-FDG PET

**DOI:** 10.1371/journal.pone.0169082

**Published:** 2017-01-04

**Authors:** Tien T. Tang, David A. Rendon, Janice A. Zawaski, Solmaz F. Afshar, Caterina K. Kaffes, Omaima M. Sabek, M. Waleed Gaber

**Affiliations:** 1 Department of Bioengineering, Rice University, Houston, Texas, United States of America; 2 Hematology-Oncology Section, Department of Pediatrics, Baylor College of Medicine, Houston, Texas, United States of America; 3 Department of Surgery, Houston Methodist Hospital Research Institute, Houston, Texas, United States of America; University of California Davis, UNITED STATES

## Abstract

Positron emission tomography using ^18^F-Fluro-deoxy-glucose (^18^F-FDG) is a useful tool to detect regions of inflammation in patients. We utilized this imaging technique to investigate the kinetics of gastrointestinal recovery after radiation exposure and the role of bone marrow in the recovery process. Male Sprague-Dawley rats were either sham irradiated, irradiated with their upper half body shielded (UHBS) at a dose of 7.5 Gy, or whole body irradiated (WBI) with 4 or 7.5 Gy. Animals were imaged using ^18^F-FDG PET/CT at 5, 10 and 35 days post-radiation exposure. The gastrointestinal tract and bone marrow were analyzed for ^18^F-FDG uptake. Tissue was collected at all-time points for histological analysis. Following 7.5 Gy irradiation, there was a significant increase in inflammation in the gastrointestinal tract as indicated by the significantly higher ^18^F-FDG uptake compared to sham. UHBS animals had a significantly higher activity compared to 7.5 Gy WBI at 5 days post-exposure. Animals that received 4 Gy WBI did not show any significant increase in uptake compared to sham. Analysis of the bone marrow showed a significant decrease of uptake in the 7.5 Gy animals 5 days post-irradiation, albeit not observed in the 4 Gy group. Interestingly, as the metabolic activity of the gastrointestinal tract returned to sham levels in UHBS animals it was accompanied by an increase in metabolic activity in the bone marrow. At 35 days post-exposure both gastrointestinal tract and bone marrow ^18^F-FDG uptake returned to sham levels. ^18^F-FDG imaging is a tool that can be used to study the inflammatory response of the gastrointestinal tract and changes in bone marrow metabolism caused by radiation exposure. The recovery of the gastrointestinal tract coincides with an increase in bone marrow metabolism in partially shielded animals. These findings further demonstrate the relationship between the gastrointestinal syndrome and bone marrow recovery, and that this interaction can be studied using non-invasive imaging modalities.

## Introduction

In the event of a nuclear disaster or accident, healthcare facilities would need to triage patients with varying degree of radiation exposure and dose. Severity of the exposure is characterized on a spectrum which includes the hematopoietic and gastrointestinal syndrome. The hematopoietic syndrome (≥2 Gy in humans) is characterized by the onset of bone marrow damage, pancytopenia, hematopoietic drainage, and immune suppression. At a higher dose (≥6 Gy in humans) patients are diagnosed with the gastrointestinal syndrome which is characterized by severe diarrhea, loss of blood and electrolytes and can ultimately lead to death 10–14 days post exposure [1]. Radiation-induced death of highly proliferative cells, hematopoietic progenitor cells and intestinal crypt stem-cell population, are the underlying phenomena in both of these syndromes [1–4]. Differentiating between survival groups across these syndromes becomes more difficult when partial body shielding occurs [5–7]. Furthermore, nuclear accidents, such as Chernobyl and Fukushima-Daiichi, highlight the urgency for developing radiomitigators against acute radiation exposure (DREARE). There has been intense efforts by the scientific community, spurred by several federal government initiatives, to investigate the mechanisms underlying DREARE and for developing medical counter measures to mitigate its deadly effects [8].

Although there are several gaps in our knowledge [5–7] of the exact relationship between shielding, and survival, it is accepted that the degree and kinetics of marrow reconstitution, the total dose received, percent of body exposed, and anatomical region irradiated or shielded are important factors in determining survival [9]. It has been established that shielding, results in substantial advantages in survival [10]. However, similar to whole body exposure, partial exposure results in a decline in the body’s blood and immune cells albeit, depending on the degree of shielding, to lesser degree with shorter time of recovery [5, 11]. In addition, Terry et al., have shown that the response of the gastrointestinal organ to radiation exposure is related to the extent of bone marrow depletion [12]. In the aftermath of a nuclear disaster the victims’ total dose and percent of body exposure is estimated from a complicated formula that takes into account geographical information, such as distance from radiation source, and obstacles or absorbing factors in the direct line between the victim and the source, as well as biological dosimetry, usually from body fluids, skin or tissue samples [13–15]. Despite the advances in biological dosimetry it cannot yet distinguish total from partial body exposure to provide proper patient care [16]. Therefore, an effective triaging paradigm that includes quick, noninvasive measurements of bone marrow as well as gastrointestinal damage is necessary to differentiate patients requiring immediate care from those who could be followed up at a later time.

Positron emission tomography (PET) is a molecular imaging technique that is commonly used in the clinic to quantify and observe different biological processes. ^18^F-Fluro-deoxy-glucose (^18^F-FDG) is a well characterized radiopharmaceutical commonly used in metabolic assessment for cancer detection and staging [17, 18]. ^18^F-FDG is a glucose analog which accumulates in tissue with high metabolic activity such as solid tumors [19]. This imaging technique can further be utilized for imaging inflammation. Immune cell activation requires an increase in glucose consumption and can therefore be detected using this imaging technique [20–22]. Radiation injury to the gastrointestinal tract results in loss of crypt intestinal stem cells and break down of the mucosal barrier leading to the activation and recruitment of immune cells such as neutrophils, macrophages and cytotoxic T-cells to the site of injury [3, 23, 24]. The localization of activated immune cells and therefore local increase in glucose consumption can be characterized using ^18^F-FDG imaging. Furthermore, radiation-induced bone marrow injury has been shown to damage highly proliferative progenitor cell populations and its vascular network [25, 26]. These functional and structural effects result in a reduction in the metabolic activity of the bone marrow [27]. Thus, in this work we hypothesize that ^18^F-FDG imaging is a tool that can be utilized to investigate the kinetics of gastrointestinal and bone marrow recovery after radiation exposure through the inflammatory response of these different regions. Despite the concerns with using nuclear medicine the quantitative information gained outweigh the risks, with significantly lower dose compared to what a patient would receive in the event of a nuclear disaster. In addition, PET imaging is already being actively used in the clinic which will allow for rapid clinical translation.[28].

In this study we will evaluate the diagnostic value of ^18^F-FDG PET/CT imaging in detecting gastrointestinal syndrome acutely after radiation exposure using a rodent model. Furthermore, we will use ^18^F-FDG PET/CT to assess the changes in metabolic activity in the bone marrow of whole body and partial body radiation exposed animals to investigate the relationship between gastrointestinal syndrome recovery and bone marrow preservation.

## Materials and Methods

### Animal irradiation

Animal studies were done in accordance with guidelines and approval from the Baylor College of Medicine Institutional Animal Care and Use Committees. 6–7 weeks old male Sprague Dawley rats (225–249 g) (Harlan Laboratories, Indianapolis, IN) were divided into four groups: a control group that received no radiation (sham), upper half shielded (UHBS) with 7.5 Gy x-ray, or whole body irradiated (WBI) at 4 or 7.5 Gy. A separate cohort (n = 2 per group) was also irradiated for histology. Animals were anesthetized with isoflurane gas (1–3%) mixed with oxygen at a gas flow rate of 1 L/minute, positioned prone and irradiated at a dose rate of 1.16 Gy/min (RS 2000 x-ray irradiator, Rad Source, GA). UHBS animals were shielded using 0.5 cm-thick piece of malleable lead covering the animal from the sternum upward.

### ^18^F-FDG PET/CT imaging

All CT and PET images were acquired using an Inveon scanner (Siemens AG, Knoxville, TN). For ^18^F-FDG PET/CT imaging, animals were fasted approximately 12 hours before injection of radioisotope. One hour prior to scanning, each animal received approximately 12.58 MBq (340 μCi) of ^18^F-FDG (Cyclotope, Houston, TX) through intravascular (IV) injection. A respiratory pad was placed under the abdomen of the animal to monitor respiration (Biovet, Newark, NJ). Animals were anesthetized with isoflurane gas (1–3%) mixed with oxygen at a flow rate of 0.5–1 L/minute, and adjusted accordingly during imaging to maintain normal breathing rates. A CT scan was acquired with the following specifications: 220 acquired projections covering 220°, a source to detector distance of 312.91 mm and a source to center rotation distance of 183.92. Each projection was 650 ms with x-ray tube voltage and current set at 80 kVp and 500 μA, respectively. A 40 minute PET scan was immediately acquired afterward. The PET scans were reconstructed using OSEM3D reconstruction method and registered to the CT scan for attenuation correction.

### PET image analysis

Using the reconstructed PET scan, the gastrointestinal tract was selected to form a region of interest (ROI) using Inveon Research Workspace (Siemens AG, Knoxville, TN). Tissue uptake is measured using standardized uptake value normalized to body weight (SUV) or radioactivity activity concentration over amount injected normalized by body weight of the animal. Bone marrow ROIs were selected using the spatial information from the CT scan and an algorithm developed in-house, the details of our method has been previously published [25]. Briefly, a mask was generated corresponding to the bone structures from the CT scan and applied to the PET data. Different ROIs were segmented out from the mask for quantification of radioactivity in the bone marrow.

### Histopathology

Small intestines were fixed in 10% buffered formalin, paraffin embedded and cut into 7 μm sections. The sections were stained with hematoxylin and eosin (H&E) using standard protocols. Villi lengths were measured using ImageJ [29]. Sections of the spine were fixed in 10% buffered formalin for 48 hours and decalcified (TBD-2 Decalcifier, Thermo Scientific, Waltham, MA) for 72 hours. Paraffin embedded blocks were cut into 4 μm sections and stained with H&E.

### Statistical analysis

The data are presented as mean ± standard deviation. A one-way analysis of variance (ANOVA) with multiple comparison was used with statistical significance set at p < 0.05. Analysis was done using GraphPad Prism (GraphPad Software, La Jolla, CA).

## Results

### Inflammation

#### ^18^F-FDG PET/CT imaging

**Fig 1:**
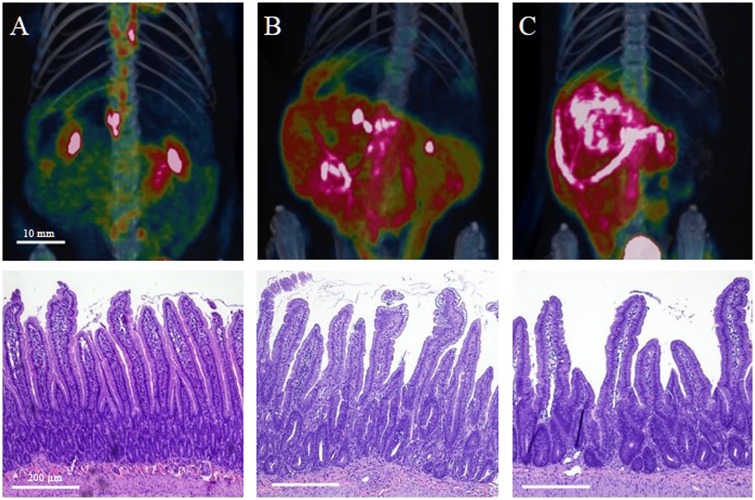
^18^F-FDG uptake of the gastrointestinal tract is greater in 7.5 Gy whole body irradiated and upper half body shielded animals when compared to sham. Reconstructed PET/CT images showing ^18^F-FDG uptake (top) and corresponding hematoxylin and eosin-stained intestine sections (bottom) of sham (A), 7.5 Gy whole body irradiated (B), and upper half body shielded (C) animals 5 days post radiation exposure.

**Fig 2:**
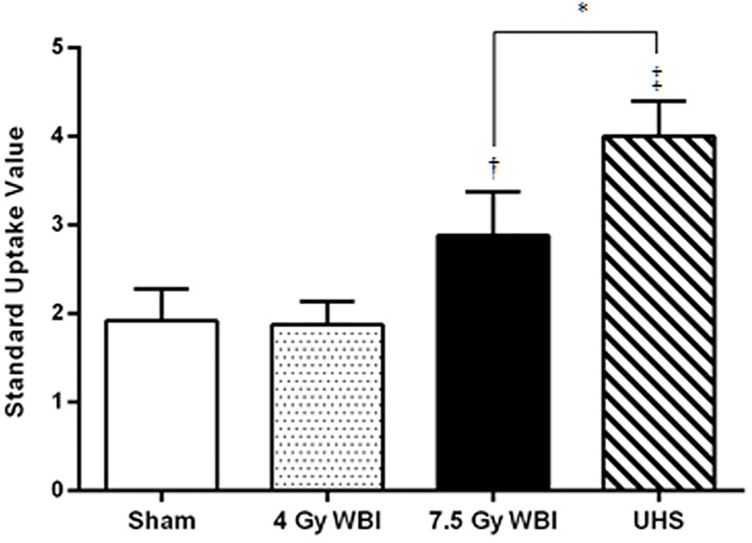
Direct radiation exposure to gastrointestinal tract can be detected with PET/CT imaging. Graph of mean standard uptake value of ^18^F-FDG in the gastrointestinal tract 5 days post irradiation. A significant increase is observed in 7.5 whole body irradiated and upper half shielded groups compared to sham group. Data shown mean ± standard deviation; *P<0.05, †P<0.01, ‡P<0.001.

^18^F-FDG PET/CT imaging can detect gastrointestinal damage due to radiation exposure (Fig 1). When compared to sham (SUV = 1.92±0.36, n = 5), irradiated animals, at a dose that induces the gastrointestinal syndrome (7.5 Gy), have increased metabolic activity 5 days after radiation exposure, regardless of shielding (SUV = 2.88±0.49, n = 6, p<0.01 7.5 Gy WBI; SUV = 4±0.4, n = 3, p<0.001 UHBS) (Fig 2). At a lower dose of 4 Gy WBI, which does not cause the gastrointestinal syndrome, ^18^F-FDG PET/CT imaging was not able to detect any significant increase in uptake compared to sham (SUV = 1.88±0.37, n = 4) at 5 days after radiation (Fig 2). In UHBS animals, this inflammatory response was sustained through day 10 (SUV = 2.6±0.36, n = 4, p<0.05) returning to values not significant from sham animals by day 35 (SUV = 2.04±0.52, n = 5) (S1 Fig). Quantification of ^18^F-FDG uptake in the gastrointestinal tract indicated a significantly higher inflammatory response (p<0.05) in UHBS (SUV = 4±0.4, n = 3) compared to WBI (SUV = 2.88±0.49, n = 6) at 5 days post irradiation (Fig 2).

### Histopathology

To corroborate our findings, H&E stained sections of the gastrointestinal tract where analyzed on days 5, 10 and 35 days post irradiation (Fig 1, S1 Fig). Compared to sham, irradiated tissue of both WBI and UHBS animals showed blunting of the villi and loss of structural integrity at day 5 (Table 1). By day 10 UHBS animals showed signs of recovery as demonstrated by the increase in villi length and the substantial decline in blunting. By 35 days the morphology of the irradiated gastrointestinal tract had returned to normal (S1 Fig). This trend of blunting and damage at early time points and recovery parallels the changes in ^18^F-FDG uptake.

**Table 1:** Villus Length at 5 Days Post irradiation.

	Sham	WBI 7.5 Gy	UHBS
Mean ± SD (μm)	515.95 ± 113.09	410.70 ± 107.49^‡^	404.31 ± 71.28 ^§^

^‡^ P<0.001 compared to sham

^§^P<0.0001 compared to sham

### Bone Marrow Activity and Gastrointestinal Recovery

#### ^18^F-FDG PET/CT imaging bone marrow

**Fig 3:**
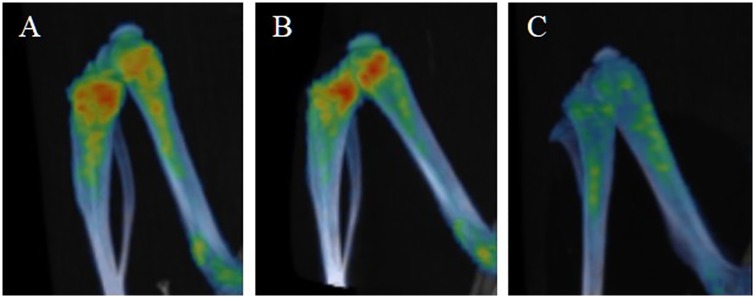
Metabolic activity in bone marrow is lower at the higher radiation dose. Reconstructed PET/CT images showing ^18^F-FDG uptake of (A) sham, (B) 4 Gy and (C) 7.5 Gy whole body irradiated animals 5 days post radiation exposure.

**Fig 4:**
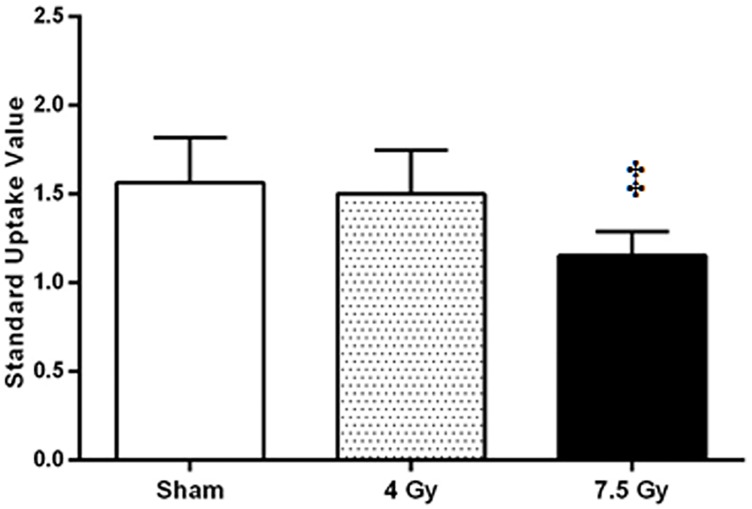
Impact of radiation on bone marrow is dose dependent. Graph of mean standard uptake value of ^18^F-FDG in the bone marrow with significant decrease in activity in 7.5 Gy whole body irradiated group compared to sham group 5 days post radiation exposure. Data shown mean ± standard deviation; ‡P<0.001.

**Fig 5:**
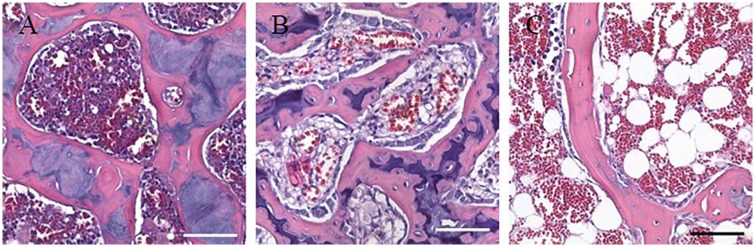
Radiation exposure leads to loss of hematopoietic cells, loss of structure and noticeable hemorrhaging with degree of damage scaling with dose. Hematoxylin and eosin images of (A) sham, (B) 4 Gy and (C) 7.5 Gy whole body irradiated animals 5 days post radiation exposure.

Radiation at a sufficiently high dose lowers bone marrow metabolic activity (Fig 3). Animals that received a gastrointestinal syndrome inducing radiation dose (7.5 Gy) had a significantly lower ^18^F-FDG uptake in the bone marrow compared to sham animals (SUV = 1.56±0.19, n = 4 sham; SUV = 1.15±0.11, n = 6, 7.5 Gy WBI, p<0.001) at 5 days post-irradiation (Fig 4). Detection of bone marrow damage using ^18^F-FDG seems to be limited by the radiation dose received or degree of damage. No significant difference was observed for animals that received 4 Gy WBI (SUV = 1.5±0.22, n = 4) compared to sham animals (Fig 4) even though histological examination indicates some depletion of progenitor cells and fat formation in the bone marrow (Fig 5). WBI at 7.5 Gy animals however exhibited much higher degree of damage to the bone marrow.

**Fig 6:**
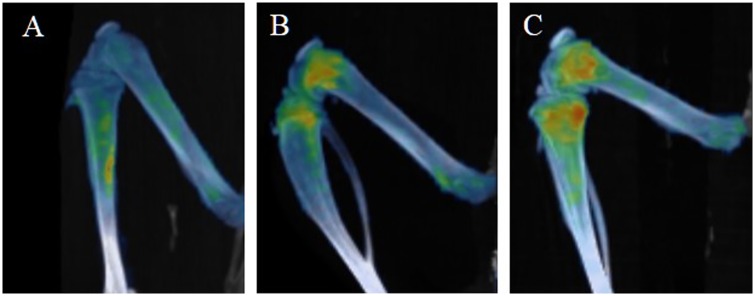
Metabolic activity in bone marrow recovers post irradiation when animals are shielded. Reconstructed PET/CT images showing ^18^F-FDG uptake of upper half body shielded animals at (A) 5 (B) 10 and (C) 35 days post radiation exposure.

**Fig 7:**
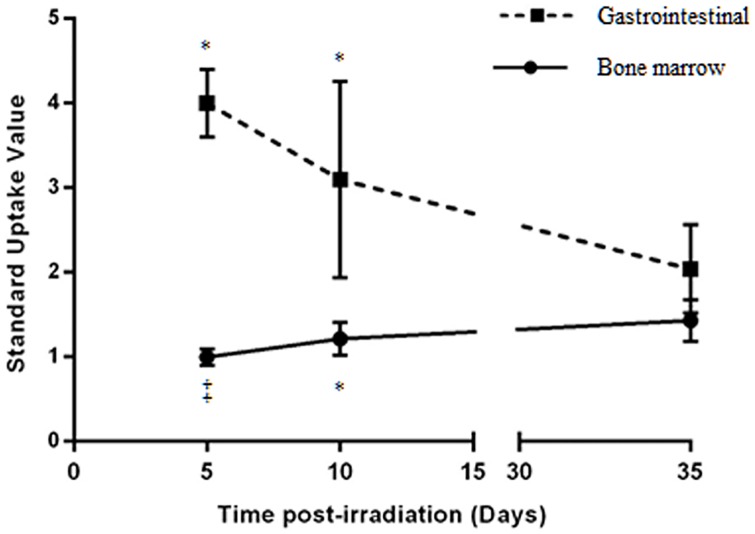
Recovery in the metabolic activity of the gastrointestinal tract coincides with that of the bone marrow activity. Graph of mean standard uptake value of ^18^F-FDG in the gastrointestinal tract and bone marrow of upper half body shielded at 5, 10 and 35 days post radiation exposure, showing full recovery of both tissues by day 35. Data shown as mean ± SD; *P<0.05, ‡P<0.001 compared to sham animals.

In order to investigate the relationship between irradiated bone marrow metabolism and recovery of the gastrointestinal syndrome we examined bone marrow activity at 5, 10 and 35 days post irradiation in UHBS animals (Fig 6). Results from the analysis indicated a full recovery in both the bone marrow (SUV = 1.43±024, n = 5) and gastrointestinal tract by day 35. ^18^F-FDG uptake in the bone marrow was significantly lower 5 days after radiation exposure and remains so up to day 10 (5 days, SUV = 1.15±0.11, n = 6 UHS, p<0.001) (10 days, SUV = 1.18±0.25, n = 3, p<0.05) compared to sham (SUV = 1.56±0.19, n = 4). The return of the bone marrow activity to sham levels parallels the recovery of the gastrointestinal tract (Fig 7).

## Discussion

In our study, we have demonstrated that ^18^F-FDG PET imaging can be used to diagnose the inflammation associated with radiation-induced gastrointestinal syndrome at an early time point and can be used to follow its recovery kinetics after the initial exposure. Our results indicate that the degree of gastrointestinal inflammation can be differentiated by ^18^F-FDG activity in which shielding led to higher uptake as early as 5 days post exposure. While histological examination did not show a noticeable damage in the UHBS group, the higher ^18^F-FDG uptake in UHBS animals compared to WBI can be a result of the shielding preserving a critical constituency of the immune cells that can still be called to the site of damage and produce a higher metabolic signal. In addition, bone marrow metabolic activity demonstrated a dose-dependent decline in which PET imaging did not detect any changes in the 4 Gy WBI but the 7.5 Gy WBI was significantly lower compared to sham at 5 days after irradiation. We also observed a steady increase in bone marrow metabolic activity after the initial exposure at days 5, 10, and 35 in UHBS animals. Most interestingly, our results show that as bone marrow metabolic activity recovers in almost a linear progression, the gastrointestinal inflammation declines in a biphasic fashion, with a sharper decline between days 5 and 10, during the same period in UHBS animals.

There have been several clinical as well as translational animal studies that utilized PET/CT imaging to investigate the use of ^18^F-FDG uptake in monitoring gastrointestinal disease [30–36]. In these studies, the imaging signal has been related to the endoscopic, colonoscopic, and histological findings. Intestinal ^18^F-FDG signal can arise from several sources: local lesions and/or immune cells at the site of inflammation. False positives, [37] or ^18^F-FDG uptake that cannot be related to clinical lesions, limits the specificity of this imaging tool [38]. Brewer et al., explained this discrepancy by suggesting that ^18^F-FDG uptake, caused by mucosal inflammation, might precede the development of such clinical lesions [34]. Louis et al., demonstrate that by using the CT anatomical localization data the specificity of the imaging increases significantly [30]. Further, the motility of the gastrointestinal tract and the energy required in such process, means that a certain base level of ^18^F-FDG uptake is variably observed, which limits the sensitivity of the imaging results. Gastroenterologists have developed several indices that incorporate clinical features, endoscopic, and laboratory markers to diagnose the gastrointestinal tract [39]. Imaging measurements could provide an extra noninvasive index in the diagnostic process.

Radiation induced damage to the crypt cells in the gastrointestinal tract elicits a cascade of inflammatory response. It has been shown that within hours of exposure crypt cells undergo apoptosis accompanied by shortening of the villi, which is observed days afterwards [40]. In addition to the morphological changes, it has also been reported that there is a shift in the local microenvironment through the recruitment of immune cells such as neutrophils, macrophages and T cells [23]. These recruited immune cells into the gastrointestinal tract could be the main source of glucose uptake that provides the signal detected through our ^18^F-FDG PET/CT imaging. This supports our results demonstrating higher uptake in the UHBS and 7.5 Gy WBI animals compared to sham animals. It has been shown that the immune cell population is greatly altered after whole body irradiation with reduction in mucosal macrophages, neutrophils and lymphocytes [24]. Further, they demonstrate that the shift in immune cell population is also influenced by the recruitment of cells from outside the irradiated field. Therefore, shielding or protection of the bone marrow will ultimately protect some of the immune cells making them more readily available to the body to call to the site of damage. However, if the gastrointestinal tract is in the field of radiation it would suffer irrespective of the amount of bone marrow shielded. Our data demonstrates this phenomenon and further shows that as more immune cells are available, as in the case in UHBS animals, there is more homing of these immune cells to the inflamed gastrointestinal tract, hence the higher metabolic signal compared to 7.5 Gy WBI animals.

In addition to gastrointestinal tract, ^18^F-FDG PET/CT imaging can also be utilized to investigate the effects of radiation on other parts of the body such as bone marrow metabolism [27, 41, 42]. Our results agrees with these findings and further demonstrate that ^18^F-FDG PET/CT imaging can detect radiation-induced metabolic changes in the event of accidental exposure, which can be critical for determining survival. Analysis of the bone marrow did not detect any significant changes in metabolism at the lower dose of 4 Gy. While it seems that this dose was sufficient to cause a noticeable reduction in progenitor cells, as demonstrated by histological examination, this cellular damage does not translate to significant impairment in metabolic function as detected by our imaging.

Preservation of bone marrow has been shown to increase survival rates after radiation exposure at doses that are within the range of the gastrointestinal syndrome (7.5 Gy in rats) [12, 25, 43] and the role of bone marrow in the recovery of the gastrointestinal syndrome have been studied and established through many studies [12, 43]. By affecting survival, partial shielding can mask the effect of the gastrointestinal syndrome, which renders our imaging observations of both bone marrow and gastrointestinal signals ever more important. Further, Saha et al. [44] have demonstrated that transplantation of marrow derived stromal cells being able to mitigate the radiation damage. The results from these previous studies agree with our findings, were we observe a return of bone marrow integrity to sham levels at around the same time point of gastrointestinal recovery and more importantly this relationship can be observed noninvasively.

## Conclusion

^18^F-FDG PET/CT imaging can be used to detect gastrointestinal damage after radiation exposure and differentiate between shielded and total body irradiation in a rodent model. We demonstrate the unique advantage that ^18^F-FDG imaging offers in diagnosing radiation-induced damage simultaneously to the gastrointestinal tract and bone marrow and the ability to non-invasively follow recovery. However, a more thorough study of the combined effect of the different permutations of dose and shielding would need to be conducted to establish the sensitivity and robustness of this imaging technique to diagnose and follow the recovery of radiation victims. Finally, our results can also be used as a model to evaluate the efficacy of radiomitagotrs that target the inflammatory pathway and follow the response in the gastrointestinal tract and bone marrow simultaneously.

## Supporting Information

S1 Fig Inflammation of the gastrointestinal tract recovers in upper half shielded animals. Reconstructed PET/CT images showing ^18^F-FDG uptake (top) and corresponding hematoxylin and eosin-stained intestine sections (bottom) of upper half shielded animals (A) 10 days and (B) 35 days post radiation exposure.
(TIF)
