# Supplementary figures and images for: Imaging Radiation-Induced Gastrointestinal, Bone Marrow Injury and Recovery Kinetics Using 18F-FDG PET
Source: PLoS One. 2017 Jan 4;12(1):e0169082. doi: 10.1371/journal.pone.0169082 (PMC5214459; doi:10.1371/journal.pone.0169082)

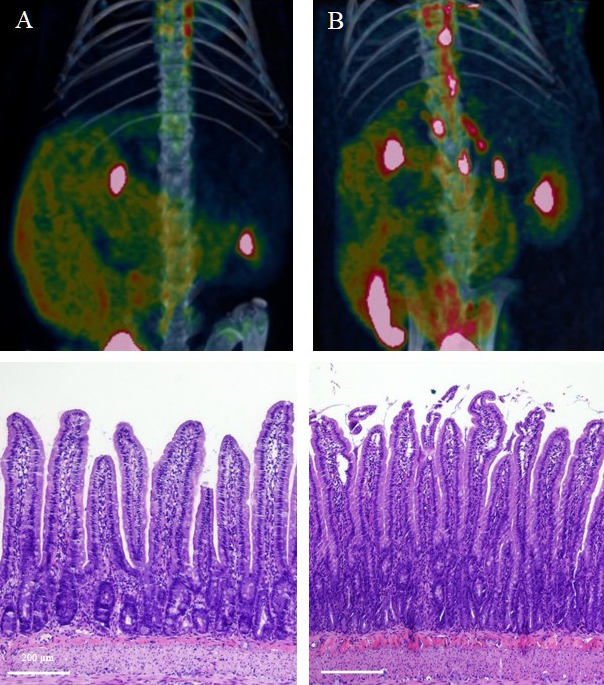

Supplement: S1 Fig — Reconstructed PET/CT images showing 18F-FDG uptake (top) and corresponding hematoxylin and eosin-stained intestine sections (bottom) of upper half shielded animals (A) 10 days and (B) 35 days post radiation exposure. (TIF) [file pone.0169082.s001.tif]
